# The association between frailty and MRI features of cerebral small vessel disease

**DOI:** 10.1038/s41598-019-47731-2

**Published:** 2019-08-05

**Authors:** Ilse M. J. Kant, Henri J. M. M. Mutsaerts, Simone J. T. van Montfort, Myriam G. Jaarsma-Coes, Theodoor D. Witkamp, Georg Winterer, Claudia D. Spies, Jeroen Hendrikse, Arjen J. C. Slooter, Jeroen de Bresser, BioCog Consortium, Franz Paul Armbruster, Axel Böcher, Diana Boraschi, Friedrich Borchers, Giacomo Della Camera, Edwin van Dellen, Ina Diehl, Thomas Bernd Dschietzig, Insa Feinkohl, Ariane Fillmer, Jürgen Gallinat, Bettina Hafen, Katarina Hartmann, Karsten Heidtke, Anja Helmschrodt, Paola Italiani, Bernd Ittermann, Roland Krause, Marion Kronabel, Simone Kühn, Gunnar Lachmann, Daniela Melillo, David K. Menon, Laura Moreno-López, Rudolf Mörgeli, Peter Nürnberg, Kwaku Ofosu, Maria Olbert, Malte Pietzsch, Tobias Pischon, Jacobus Preller, Jana Ruppert, Reinhard Schneider, Emmanuel A. Stamatakis, Simon Weber, Marius Weyer, Stefan Winzeck, Alissa Wolf, Fatima Yürek, Norman Zacharias

**Affiliations:** 1 Department of Intensive Care Medicine and Brain Center Rudolf Magnus, UMC Utrecht, Utrecht University, Heidelberglaan 100, Utrecht, The Netherlands; 2 Department of Radiology and Brain Center Rudolf Magnus, UMC Utrecht, Utrecht University, Heidelberglaan 100, Utrecht, The Netherlands; 3 Department of Radiology, Leiden University Medical Center, Leiden, The Netherlands; 4 Experimental and Clinical Research Center (ECRC), Charité – Universitätsmedizin Berlin, Berlin, Germany; 5 PharmaImage Biomarker Solutions GmbH, Berlin, Germany; 6 Department of Anesthesiology and Operative Intensive Care Medicine (CCM,CVK), Charité – Universitätsmedizin Berlin, and corporate member of Freie Universität Berlin, Humboldt-Universität zu Berlin, and Berlin Institute of Health, Berlin, Germany; 7 Immundiagnostik AG, Stubenwald-Allee 8a, 64625 Bensheim, Germany; 8 National Research Council, Institute of Protein Biochemistry, Napoli, Italy; 9 Molecular Epidemiology Research Group, Max-Delbrück-Center for Molecular Medicine in the Helmholtz Association (MDC), Berlin, Germany; 10 Physikalisch-Technische Bundesanstalt (PTB), Braunschweig and Berlin, Braunschweig, Germany; 11 Clinic and Policlinic of Psychiatry and Psychotherapy, University Medical Center, Hamburg-Eppendorf, Germany; 12 ATLAS Biolabs GmbH, Berlin, Germany; 13 University of Luxembourg, Luxembourg Centre for Systems Biomedicine, Luxembourg, Luxembourg; 14 Clinic and Policlinic of Psychiatry and Psychotherapy, University Medical Center, Hamburg-Eppendorf, Lise Meitner Group for Environmental Neuroscience, Max Planck Institute for Human Development, Berlin, Germany; 15 Division of Anaesthesia, Department of Medicine, University Of Cambridge, Cambridge, UK; 16 Cellogic GmbH, Niedstrasse 21, 12159 Berlin, Germany; 17 Cambridge University Hospitals NHS trust, Addenbrooke’s Hospital, Cambridge, UK; 18 Department of Psychiatry and UMC Utrecht Brain Center, University Medical Center Utrecht, Utrecht University, Utrecht, The Netherlands

**Keywords:** Geriatrics, Brain, Cerebrovascular disorders, Outcomes research

## Abstract

Frailty is a common syndrome in older individuals that is associated with poor cognitive outcome. The underlying brain correlates of frailty are unclear. The aim of this study was to investigate the association between frailty and MRI features of cerebral small vessel disease in a group of non-demented older individuals. We included 170 participants who were classified as frail (n = 30), pre-frail (n = 85) or non-frail (n = 55). The association of frailty and white matter hyperintensity volume and shape features, lacunar infarcts and cerebral perfusion was investigated by regression analyses adjusted for age and sex. Frail and pre-frail participants were older, more often female and showed higher white matter hyperintensity volume (0.69 [95%-CI 0.08 to 1.31], p = 0.03 respectively 0.43 [95%-CI: 0.04 to 0.82], p = 0.03) compared to non-frail participants. Frail participants showed a non-significant trend, and pre-frail participants showed a more complex shape of white matter hyperintensities (concavity index: 0.04 [95%-CI: 0.03 to 0.08], p = 0.03; fractal dimensions: 0.07 [95%-CI: 0.00 to 0.15], p = 0.05) compared to non-frail participants. No between group differences were found in gray matter perfusion or in the presence of lacunar infarcts. In conclusion, increased white matter hyperintensity volume and a more complex white matter hyperintensity shape may be structural brain correlates of the frailty phenotype.

## Introduction

Frailty is a chronic condition of increased vulnerability to physiological stressors that is common in older individuals and is most often described using the physical frailty phenotype^1–3^. Frail older individuals have a higher risk of falling, postoperative complications, dependency and cognitive decline, compared to non-frail counterparts^1,4,5^. The impact of these consequences of frailty on a societal and economic level in an aging population stresses the importance of investigating the pathways leading to a frail condition. To date, the biological pathways leading to the decline of multiple physiological systems remain unknown. The association between frailty and cognitive disorders suggests a structural brain correlate, but this is currently largely unknown.

Cerebral small vessel disease (SVD) is a syndrome that is characterized by manifestations of diseases of the small vessels in the brain, with probably different underlying pathophysiology and etiology^6,7^. SVD is one of the major causes of stroke, cognitive decline and dementia in older individuals^6,8^. The most commonly used MRI features of SVD are white matter hyperintensity of presumed vascular origin (WMH) volume and the presence of lacunar infarcts^6,9^.

Previous studies on the association between of frailty with WMH volume or lacunar infarcts have shown inconsistent results^10–16^. Due to the heterogeneous nature of SVD, other MRI features of SVD may provide additional information^17–20^. Recent studies have shown that WMH shape is a novel promising feature related to more severe small vessel changes^19,20^. Furthermore, cerebral perfusion measured by arterial spin labeling (ASL) MRI could detect early hemodynamic changes that may be related to SVD^21–23^. Frailty may therefore be associated with both structural brain changes of SVD as well as quantitative hemodynamic changes.

The aim of this study was to investigate the association between frailty and MRI features of cerebral SVD in a group of non-demented older individuals who were scheduled for major elective surgery. We assessed both commonly used structural features (WMH volume and presence of lacunar infarcts) and novel structural (WMH shape) and hemodynamic features (cerebral perfusion) of cerebral SVD.

## Results

Of the 178 participants that completed the MRI scanning protocol, a total of 8 participants had to be excluded from all analyses, due to extremely large ventricles that hindered accurate segmentation (n = 1), an incomplete FLAIR and ASL sequence (n = 1), or major MRI artifacts (e.g. motion; n = 6), leaving 170 participants for the current study. Demographics for frail (n = 30), pre-frail (n = 85) and non-frail (n = 55) participants are shown in Table 1. Frail participants were older, more often female, had higher ASA classification scores and a higher body mass index (BMI) compared to pre- and non-frail individuals. No between-group differences in other vascular risk factors or MMSE scores were found. A total of 13 participants had to be excluded from the WMH feature analyses due to major segmentation errors (n = 6), FLAIR artifacts (e.g. motion, n = 4) or no availability of a FLAIR sequence (n = 2). A total of 90 participants had an ASL image of sufficient quality for perfusion analysis, and a total of 156 participants had an ASL image that could be used for spatial CoV analysis of vascular signal.

**Table 1:** Demographics.

	Frail (n = 30)	Pre-frail (n = 85)	Non-frail (n = 55)	p-value
Age	74 ± 5	72 ± 5	70 ± 4	0.01
Female gender	16 (53%)	24 (28%)	12 (22%)	0.01
MMSE	28 (28, 29)	29 (27, 30)	29 (28, 30)	0.81
Depressive symptoms	4 (13%)	6 (7%)	1 (2%)	0.12
ASA score				0.04
I	1 (3%)	8 (9%)	11 (20%)	
II	15 (50%)	45 (53%)	32 (58%)	
III	14 (47%)	32 (38%)	12 (22%)	
Vascular risk factors				
Diabetes	8 (27%)	12 (14%)	6 (11%)	0.13
BMI	29 ± 6	27 ± 4	26 ± 4	0.01
Obesity	9 (30%)	19 (22%)	6 (11%)	0.08
Hypertension	17 (57%)	45 (53%)	22 (40%)	0.21
Hyperlipidemia	12 (40%)	36 (42%)	16 (29%)	0.28
Current smoker	3 (10%)	8 (10%)	3 (5%)	0.60
TIA/CVA	3 (10%)	5 (6%)	1 (2%)	0.10
Frailty components				
Slowness	23 (77%)	18 (21%)	—	n/a
Weakness	18 (60%)	27 (32%)	—	
Weight loss	11 (37%)	19 (22%)	—	
Exhaustion	24 (80%)	22 (26%)	—	
Mobility	27 (90%)	34 (40%)	—	

Note. Data represent n (percentage), mean ± SD or median (interquartile range). A one-way ANOVA comparison of three groups was performed on continuous data. A chi-square comparison of three groups was performed for categorical data.

MMSE: mini-mental state exam. ASA: classification of disease severity for the American Society of Anesthesiologists. BMI: body-mass index. TIA: transient ischemic attack. CVA: cerebrovascular accident.

### WMH volume

Frail participants showed a higher total (0.69 [95% CI 0.08 to 1.31]), p = 0.03 and periventricular/confluent (0.67 [95% CI 0.06 to 1.30], p = 0.03) natural log transformed WMH volume compared to non-frail participants (see Table 2). Pre-frail participants showed also a higher total natural log transformed WMH volume (0.43 [95% CI 0.04 to 0.81], p = 0.03) and a higher periventricular and confluent natural log transformed WMH volume (0.43 [95% CI 0.04 to 0.81], p = 0.03) than non-frail participants.

**Table 2:** The association between physical frailty and WMH volume.

	Frail (n = 28)	Pre-frail (n = 77)	Non-frail (n = 52)	Frail vs. non-frail	Pre-frail vs. non-frail
Total WMH volume	10.92 ± 15.80	8.68 ± 11.28	4.89 ± 7.38	0.69 (0.08, 1.31)*	0.43 (0.04, 0.82)*
Periventricular and confluent WMH volume	10.52 ± 15.68	8.32 ± 11.09	4.64 ± 7.13	0.67 (0.06, 1.3)*	0.43 (0.04, 0.81)*
Deep WMH volume	0.40 ± 0.66	0.36 ± 0.65	0.25 ± 0.49	0.55 (−0.35, 1.46)	0.27 (−0.34, 0.87)

Note. Data are represented as mean WMH volume (ml) ± SD. The linear regression analyses were adjusted for age, gender and ICV. Regression beta coefficients are presented with a 95% confidence interval. WMH volumes were multiplied by 100 and natural log transformed before performing regression analyses. *p = 0.03.

### Presence of lacunar infarcts

In total, 20% (n = 6) of the frail participants, 27% (n = 23) of the pre-frail participants and 22% (n = 12) of the non-frail participants had lacunar infarcts. Logistic regression analyses corrected for age and sex showed no between-group differences in the presence of lacunar infarcts (frail versus non-frail: OR (95% CI) = 1.25 [0.35 to 4.42], p = 0.73; pre-frail versus non-frail: OR (95% CI) = 1.35 [0.59 to 3.08], p = 0.48).

### WMH shape features

Frail participants showed a non-significant trend for a more complex shape of periventricular and confluent WMH (concavity index (0.05 [95% CI 0.00 to 0.11], p = 0.06) compared to non-frail participants (see Table 3). Pre-frail participants showed a more complex shape of periventricular and confluent WMH (a higher concavity index of 0.04 [95% CI 0.03 to 0.08], p = 0.03 and a higher fractal dimension of 0.07 [95% CI 0.00 to 0.15], p = 0.05) compared to non-frail participants (see Table 3). No between-group differences were found in shape features of deep WMH (eccentricity and fractal dimensions). In secondary analyses, the between group differences in the shape of periventricular and confluent WMH attenuated after additional correction for natural log transformed WMH volume, indicating that these differences were also partly explained by WMH volume (see supplementary Table B).

**Table 3:** The association between physical frailty and WMH shape features.

	Frail (n = 28)	Pre-frail (n = 77)	Non-frail (n = 52)	Frail vs. non-frail	Pre-frail vs. non-frail
**Periventricular/confluent WMH**					
Solidity^a^	0.29 ± 0.18	0.31 ± 0.20	0.36 ± 0.20	−0.14 (−0.43, 0.15)	−0.16 (−0.37, 0.04)
Convexity	1.15 ± 0.18	1.14 ± 0.18	1.17 ± 0.17	−0.04 (−0.13, 0.05)	−0.02 (−0.08, 0.05)
Concavity index	1.13 ± 0.13	1.13 ± 0.24	1.08 ± 0.09	0.05 (0.00, 0.11)	0.04 (0.03, 0.08)*
Fractal dimension	1.68 ± 0.26	1.67 ± 0.22	1.57 ± 0.22	0.08 (−0.03, 0.20)	0.07 (0.00, 0.15)*
**Deep WMH**					
Eccentricity	0.58 ± 0.15	0.56 ± 0.18	0.58 ± 0.10	0.01 (−0.06, 0.08)	−0.01 (−0.08, 0.05)
Fractal dimension	1.83 ± 0.20	1.81 ± 0.35	1.88 ± 0.23	−0.04 (−0.17, 0.09)	−0.06 (−0.19, 0.07)

Note. Data are represented as means ± SD. Regression analysis were adjusted for age and gender. Regression beta coefficients are presented with a 95% confidence interval. ^a^Solidity was multiplied by 100 and natural log transformed. *Concavity index: p = 0.03, Fractal dimension: p = 0.05.

### Perfusion

Analysis of global cerebral perfusion of gray matter and white matter showed no significant differences between frail and non-frail participants, and no significant differences between pre-frail and non-frail participants. Furthermore, no between group differences were found in spatial CoV of the perfusion images (see Table 4).

**Table 4:** The association between physical frailty and cerebral perfusion.

	Frail (n = 13)	Pre-frail (n = 40)	Non-frail (n = 37)	Frail vs. non-frail	Pre-frail vs. non-frail
Gray matter perfusion	97 ± 24	82 ± 17	85 ± 20	12.3 (−2.8, 27.5)	−5.3 (−18.0, 7.3)
Deep WM perfusion	28 ± 8	26 ± 10	25 ± 8	1.3 (−4.6, 7.1)	0.7 (−3.8, 5.1)
Spatial CoV^a^	2.51 ± 0.70	2.52 ± 0.61	2.36 ± 0.56	0.18 (−0.16, 0.51)	0.15 (−0.07, 0.36)

Note. Data are represented as mean ± SD. Linear regression analysis were adjusted for age and gender. Regression beta coefficients are presented with a 95% confidence interval. ^a^Data represents the spatial coefficient of variation of n = 15 frail, n = 67 pre-frail and n = 48 non-frail individuals.

### Exploratory analysis of relevant MRI features of SVD per frailty component

Exploratory analyses were performed of the MRI features of SVD that showed between-group differences (total WMH volume, concavity index, fractal dimensions), see Supplementary Table C. The physical frailty component slowness showed an association with WMH volume (0.65 [95% CI 0.23 to 1.06], p = 0.003) and with a more complex shape of WMH (fractal dimensions: 0.12 [95% CI 0.04 to 0.21], p = 0.003). The component exhaustion also showed a relation with WMH volume (0.41 [95% CI 0.00 to 0.82], p = 0.048). Other physical frailty components (weakness, weight loss, mobility) showed no significant association with the studied SVD features.

## Discussion

In summary, we observed that frail and pre-frail participants had a higher WMH volume compared to non-frail participants. Furthermore, pre-frail participants showed a more complex shape of periventricular and confluent WMH compared to non-frail participants. No between group differences were found in shape features of deep WMH, cerebral perfusion or presence of lacunar infarcts.

Previous community-based studies showed an inconsistent association between frailty and WMH volume and lacunar infarcts^10–16^. Direct comparison with these studies is however hindered by the use of different methods to assess frailty. The frailty assessment of these studies differed between the physical frailty phenotype by Fried *et al*.^11,12,14–16^, and frailty scores that included measures of cognition, such as the Edmonton Frail Scale or the frailty index^2,10,13,24^. Four of the previous studies that assessed WMH showed an association between WMH and frailty^10,11,15,16^, three other studies that were performed only found a weak or no association^12–14^. Almost all previous studies were community-based studies performed in older adults^10–13,15,16^, although the studies that did not find an association included slightly younger individuals, possibly explaining the different findings^12,13^. Our study adds to the body of evidence for an association between frailty and WMH volume^10,12,15^.

Comparison of previous studies regarding the association between frailty and lacunar infarcts is difficult due to use of different definitions of lacunar infarcts. Most previous studies did not conform with the size definition of lacunar infarcts (3–15 mm) according to the internationally accepted guidelines that we used on MRI markers of small vessel disease proposed in the STRIVE criteria^25^. One previous study found an association between frailty and cerebral infarcts >3 mm^15^, whereas another earlier study showed no association with a frailty score that included cognitive tests and infarcts <20 mm^10^. Another previous study that investigated the relation between physical performance (e.g. gait, walking speed) and lacunar infarcts in memory clinic patients showed no relation between impaired physical performance and the presence of lacunar infarcts according to the STRIVE criteria^26^. Our findings are in accordance with these previous findings, as we did not find an association between frailty and lacunar infarcts^10^. The prevalence of lacunar infarcts in our study (24% in a population with a mean age of 71 ± 5 years of age) is comparable to the prevalence in a general older population (20% in a population with a mean age of 70 ± 7 years of age)^27^.

No previous study has been performed on the association between frailty and WMH shape features, and only few studies have been performed on WMH shape features in general^19,20^. A previous cohort study in patients with symptomatic atherosclerotic disease showed that the presence of lacunar infarcts was related to a more complex WMH shape^20^. Furthermore, WMH shape was different in patients with type 2 diabetes mellitus (a condition associated with cerebral SVD) compared to healthy individuals^19^. These investigations indicate that WMH shape may be helpful in examining the heterogeneity of cerebral SVD and that a more complex shape of WMH may be associated with more severe manifestations of cerebral SVD^19,20^. Although our study is the first to show the relation between frailty and a more complex shape of WMH, these associations were not completely independent of WMH volume. A more complex WMH shape combined with increased WMH volume could be part of the structural brain changes that underlie the physical frailty phenotype.

Cerebral perfusion as measured by arterial spin labeling MRI is an indicator of brain metabolism and is one of the early markers for cognitive deterioration. Recently it has been suggested that low perfusion is a marker for SVD, although it is not yet clear whether this precedes or follows the presence of WMH^28^. Previous cross-sectional studies on SVD in both demented and non-demented populations showed that a higher WMH volume is associated with lower total CBF, although this was partly driven by co-occurrence of neurodegenerative diseases^29,30^. Our study is, to the best of our knowledge, the first that assessed the relation between global gray matter perfusion and frailty. We did not find an association between cerebral perfusion and physical frailty. A possible explanation for not finding this association may be that altered brain hemodynamics could be a marker for a more severe type of SVD in comparison to our population^29^.

No previous studies on frailty and markers of SVD additionally explored the relation between individual frailty components and features of SVD. However, some previous investigations were performed on separate components without exploring overall frailty, such as gait speed^31^, the short physical performance battery^32^, mobility^33^, and exhaustion^34^. These studies all showed an association between these components and MRI markers for SVD^31–34^. In exploratory analyses in our study, all components were associated with a higher WMH volume and a more complex WMH shape, although only slowness and exhaustion reached statistical significance. For slowness, this finding is in line with previous findings on gait speed and the short physical performance battery^31,32^. The exhaustion component in our study was derived from one question of a questionnaire on anxiety and depression, and may not be accurate enough. The recommended assessment of exhaustion is by the CES-D depression scale, which could be more sensitive and specific to exhaustion^35^. A possible explanation for the relation between exhaustion and WMH volume might be that exhausted participants were slightly more depressed, which has previously been related to a higher WMH volume^33^. As this was a secondary analysis and there is not much previous evidence, these results should be carefully interpreted.

Strengths of our study include the detailed assessment of both commonly used features of cerebral SVD (WMH volume and lacunar infarcts) and more novel features of cerebral SVD (WMH shape features and cerebral perfusion) together with a detailed assessment of the physical frailty phenotype. Limitations of our study could be the limited number of physically frail individuals, causing a difference in group size between frail, pre-frail and non-frail individuals. Due to these differences, we had a lower statistical power to detect differences between frail and non-frail individuals. This may explain why we did not find a statistically significant difference in WMH shape or perfusion between frail and non-frail individuals. Another limitation is that participants from our study were all scheduled for major elective surgery and may not be comparable to the general population. This could limit the generalizability of our findings to the general population. A limitation in our assessment of slowness is that we did not consider comorbidities such as arthritis and pulmonary disease, which could have influenced the results of walking speed and exhaustion. A technical limitation could be that WMH segmentation methods in general have a limited accuracy for segmentation of especially smaller deep WMH. This may have underestimated possible associations between frailty and shape of deep WMH.

In conclusion, increased WMH volume and a more complex shape of WMH may be structural correlates of the physical frailty phenotype.

## Methods

### Study design and participants

Data were obtained from the BioCog consortium study: an international observational study that aims to identify biomarkers of postoperative cognitive disorders^36^. The BioCog study is performed at the Charité Universitätsmedizin Berlin and the University Medical Center Utrecht^36^. The present study uses patient data that were collected at the University Medical Center Utrecht only. Patients who were included in BioCog (1) were at least 65 years of age, (2) were scheduled for major elective surgery of at least 60 minutes, and (3) had a mini-mental state exam (MMSE) score of 24 or higher. The medical ethics committee of both centers approved all procedures under ethical approval number EA2/092/14 (Berlin: Ethikkommission der Charité) and 14-469 (Utrecht: Medisch Ethische Toetsingscommissie Utrecht). All participants signed informed consent. All methods were performed in accordance with all relevant guidelines and regulations that apply to research with human participants.

### Procedure

Trained research personnel collected demographic data, data on vascular risk factors (i.e. questionnaires and medical history records, vascular risk factors, obesity, which was defined as a body-mass index of ≥30^37^) and administered the MMSE during a visit prior to surgery. A hospital anxiety and depression scale (HADS) score on the depression subscale ≥8 was seen as having depressive symptoms^38^. Anesthesiologists (in training) performed the classification for the American Society of Anesthesiologists score.

### Frailty assessment

Participants were classified based on five frailty components according to the Fried frailty phenotype^2,39^: slowness, weakness, weight loss, exhaustion and mobility. Slowness was measured by the timed up and go test^40^; when this took over 10 seconds it was scored as slow. Weakness was assessed by low maximal hand grip strength, adjusting for sex and body mass index^2^. Weight loss was determined by a self-reported unintentional weight loss of ≥5% or ≥3 kg in the previous year. Exhaustion was determined by self-reported exhaustion in the geriatric depression scale or the hospital anxiety and depression scale. Mobility was scored by a self-reported inability to walk without difficulty from the EuroQOL five dimensions questionnaire^41^ and the Barthel index^42^. Participants who did not show presence of any frailty components were classified as non-frail, those who showed presence of one or two frailty components were classified as pre-frail, and those who scored positive on three or more frailty components were classified as frail (see^14^ for more details).

### MRI scans

Participants were scanned on a Philips Achieva 3T MRI scanner. The MRI scanning protocol consisted of a three-dimensional (3D) T1-weighted sequence (voxel size = 1.0 × 1.0 × 1.0 mm^3^; TR/TE = 7.9/4.5 ms), a 3D fluid-attenuated inversion recovery (FLAIR) sequence (voxel size = 1.11 × 1.11 × 0.56 mm^3^; TR/TE/TI = 4800/125/1650 ms), a pseudo-continuous arterial spin labeling (pCASL) sequence (voxel size = 3.0 × 3.0 × 7.0 mm^3^; TR/TE = 3919/17 ms, label duration = 1650 ms, post labeling delay = 1525 ms) and a diffusion-weighted image (DWI) (voxel size = 0.96 × 1.19 × 4 mm^3^; TR/TE = 3294/68 ms). Presence of lacunar infarcts was visually rated on the T1-weighted, FLAIR and DWI images by two experienced neuro-radiologists (JB (11 years of experience) and TW (25 years of experience)) according to the standards for reporting vascular changes on neuroimaging (STRIVE) criteria^25^.

### Quantification of WMH volume and shape features

**Figure 1:**
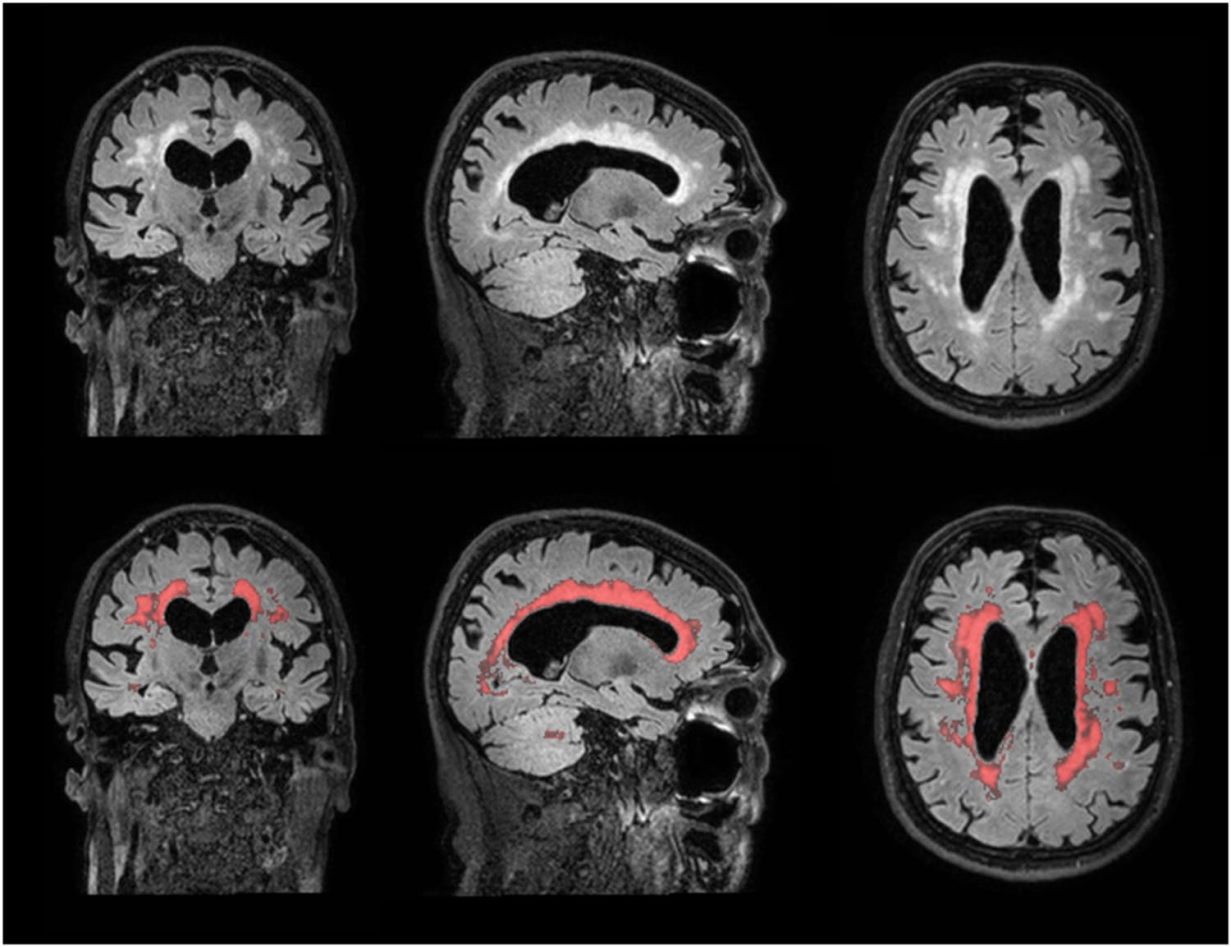
Example of a participant with a high WMH volume and complex WMH shape (left: original 3D FLAIR image; right: FLAIR image with overlay of the segmented WMH probability map in red).

3D FLAIR images were registered to the T1-weighted images using statistical parametric mapping version 12 (SPM12; Wellcome Institute of Neurology, University College London, UK, http://www.fil.ion.ucl.ac.uk/spm/doc/) for Matlab (The MathWorks, Inc., Natick, Massachusetts, United States). WMH segmentations were performed on the registered 3D FLAIR images by the lesion prediction algorithm (Schmidt, 2017, Chapter 6.1^43^) of the lesion segmentation toolbox version 2.0.15 (www.statistical-modeling.de/lst.html) for SPM12. Visual quality control for the WMH segmentations was performed by a trained researcher (IK) and supervised by a neuro-radiologist (JB), see Fig. 1 for an example. Cortical infarcts were manually delineated by a trained researcher (IK) and removed from the WMH probability maps. Lateral ventricular segmentation on the T1-weighted images was performed using the automated lateral ventricle delineation toolbox (ALVIN) in SPM8. The probabilistic WMH segmentations were thresholded at 10%. WMH within 10 mm from the lateral ventricles into the white matter were considered periventricular WMH. WMH that extended from periventricular to more than 10 mm into the deep white matter were considered confluent WMH. WMH that were located >10 mm from the lateral ventricles were considered deep WMH. WMH shape features were calculated from the thresholded WMH segmentations^20^. The solidity, convexity, concavity index and fractal dimension of periventricular and confluent WMH were calculated by reconstruction of the convex hull, volume and surface area of all individual lesions. The eccentricity and fractal dimension were calculated of deep WMH (see^20^ and Supplementary Table A for more details). For all WMH shape features, mean values per feature were calculated per patient and used for further analyses.

### CBF quantification

ASL images were processed with ExploreASL^44^. Lesion filling of the T1-weighted images was performed by the lesion segmentation toolbox version 2.0.15 (www.statistical-modeling.de/lst.html) for SPM12. The filled T1-weighted images were segmented using CAT12^45^. CBF images were motion corrected and registered to the gray matter partial volume maps^44^. The CBF images were quantified with a single compartment model^46^, after which the mean CBF was obtained for a total gray matter perfusion and deep WM region-of-interest (ROI)^47^. The spatial coefficient of variation (spatial CoV) was calculated within the total GM as a proxy parameter of vascular sufficiency (for more details see^23^). All perfusion images were rated as images containing (1) CBF contrast, (2) vascular contrast or no contrast by a trained researcher (IK), supervised by an experienced ASL researcher (HM, 7 years of experience). Images that were classified as CBF contrast were used in the perfusion analysis. Images with vascular contrast were included in the analysis of spatial CoV, but excluded from the perfusion analysis. The no contrast images that contained noise only or large artifacts were excluded from further analysis.

### Statistical analysis

For demographic analyses, three groups (frail, pre-frail and non-frail) were compared by a one-way ANOVA or chi-square test depending on the type of variable (i.e. continuous or categorical). WMH volumes, WMH shape features and cerebral perfusion were compared between the frail and non-frail group and between the pre-frail and non-frail group by linear regression analyses adjusting for age and sex. WMH volumes were natural log transformed before linear regression analysis and additionally adjusted for intracranial volume (ICV). A Pearson’s correlation coefficient was computed to assess the relation between natural log transformed total WMH volume and global cerebral gray and white matter perfusion. The presence of lacunar infarcts was compared between the frail and non-frail group and between the pre-frail and non-frail group by logistic regression analyses adjusted for age and sex.

In secondary analyses, WMH shape features were compared between groups by linear regression analyses additionally adjusted for WMH volume, to test if the found associations were WMH volume independent. Exploratory post-hoc analyses of the relation between MRI features of SVD (that showed significant between group differences) and frailty components were performed by linear regression analyses adjusted for age and sex, and for the WMH volume analysis additionally adjusted for ICV. All analyses were performed in SPSS version 25. A p-value of below 0.05 was considered statistically significant.

## Supplementary information

The association between frailty and MRI features of cerebral small vessel disease – supplementary material

## Data Availability

The datasets generated and analyzed during the current study are not publicly available as this is a substudy of a still ongoing consortium study, but may be available from the corresponding author on reasonable request
