# Supplementary material for: The association between frailty and MRI features of cerebral small vessel disease
Source: Sci Rep. 2019 Aug 5;9:11343. doi: 10.1038/s41598-019-47731-2 (PMC6683288; doi:10.1038/s41598-019-47731-2)
Supplement: Supplementary file 1 — The association between frailty and MRI features of cerebral small vessel disease – supplementary material [file 41598_2019_47731_MOESM1_ESM.docx]

**The association between frailty and MRI features of cerebral small vessel disease – supplementary material**

Ilse M.J. Kant MSc^1,2^, Henri J.M.M. Mutsaerts MD PhD^2^, Simone J.T. van Montfort MSc^1^, Myriam G. Jaarsma-Coes MSc ^3^, Theo D. Witkamp MD^2^, Georg Winterer MD PhD^4,5,6^, Claudia Spies MD PhD^6^, Jeroen Hendrikse MD PhD^2^, Arjen J.C. Slooter MD PhD^1^, Jeroen de Bresser MD PhD^2,3^

on behalf of the BioCog Consortium, clinicaltrials.gov identifier: NCT02265263, ethical approval number EA2/092/14 (Berlin), 14-469 (Utrecht)

**Authors and institutions**

1. Department of Intensive Care Medicine and Brain Center Rudolf Magnus, UMC Utrecht, Utrecht University, Heidelberglaan 100, Utrecht, The Netherlands. E: [i.kant-2@umcutrecht.nl](mailto:i.kant-2@umcutrecht.nl), [s.j.t.vanmontfort-2@umcutrecht.nl](mailto:s.j.t.vanmontfort-2@umcutrecht.nl), [a.slooter-3@umcutrecht.nl](mailto:a.slooter-3@umcutrecht.nl)
2. Department of Radiology and Brain Center Rudolf Magnus, UMC Utrecht, Utrecht University, Heidelberglaan 100, Utrecht, The Netherlands. E: [henkjanmutsaerts@gmail.com](mailto:henkjanmutsaerts@gmail.com), [j.hendrikse@umcutrecht.nl](mailto:j.hendrikse@umcutrecht.nl)
3. Department of Radiology, Leiden University Medical Center, Leiden, The Netherlands. E: [j.h.j.m.de_bresser@lumc.nl](mailto:j.h.j.m.de_bresser@lumc.nl) ; [m.g.jaarsma@lumc.nl](mailto:m.g.jaarsma@lumc.nl)
4. Experimental and Clinical Research Center (ECRC), Charité – Universitätsmedizin Berlin, Berlin, Germany
5. PharmaImage Biomarker Solutions GmbH, Berlin, Germany
6. Department of Anesthesiology and Operative Intensive Care Medicine (CCM,CVK), Charité – Universitätsmedizin Berlin, and corporate member of Freie Universität Berlin, Humboldt-Universität zu Berlin, and Berlin Institute of Health, Berlin, Germany, E: [georg.winterer@charite.de](mailto:georg.winterer@charite.de), [Claudia.spies@charite.de](mailto:Claudia.spies@charite.de)

Corresponding author: Ilse Kant, [i.kant-2@umcutrecht.nl](mailto:i.kant-2@umcutrecht.nl), 0031 – 88 75 57825

Supplementary table A: description of WMH shape features (also see^2^).

| **Name** | **Definition** | **Explanation of the formula** | **Interpretation** |
| --- | --- | --- | --- |
| Convexity (C) | $C =\frac{Convex hull area}{\mathrm{Area}}$ | Area: the surface area of the WMH lesion. Convex hull area: the smallest convex hull that fits the WMH lesion | A description of roughness; a surface with more concavities has a high area. A lower convexity is associated with a more complex shape. |
| Solidity (S) | $S =\frac{\mathrm{Volume}}{Convex hull volume}$ | Volume is the WMH volume. Convex hull volume is the volume of the smallest convex hull that fits the WMH lesion | A description of roughness; a volume with more concavities has a lower volume within the convex hull and thus a lower solidity. A lower solidity is associated with a more complex shape. |
| Concavity index (CI) | $CI= \sqrt{{(2-C)}^{2}+{(1-S)}^{2}}$ | C: Convexity; S: Solidity | A combined measure for convexity and solidity. A higher CI is associated with a more irregular or complex shape. |
| Fractal dimension (FD) | $FD= \lim_{r\to x_{\mathrm{xyz}}} \frac{\log\left( n_{r} \right)}{\log\left( \frac{1}{r} \right)}$ | N: number of boxes $x_{\mathrm{xyz}}$: voxel size  r: box size | Fractal dimension is calculated by a box counting method. A higher fractal dimension represents a more irregular shape. |
| Eccentricity (E) | $E=\frac{Minor axis}{Major axis}$ | Major axis: largest diameter of the WMH in three dimensions. Minor axis: smallest diameter orthogonal to the major axis. | A high eccentricity represents a round shape, while a low eccentricity represents a more oval shape. A low eccentricity therefore relates to “outstretched” or elongated WMH. |

Supplementary table B: Exploratory analyses of the association between frailty and WMH shape features additionally corrected for WMH volume

|  | **Frail (n=28)** | | **Pre-frail (n=77)** | **Non-frail (n=52)** | **Frail vs. non-frail (Beta (95% CI))** | **Pre-frail vs. non-frail (Beta (95% CI))** |
| --- | --- | --- | --- | --- | --- | --- |
| Periventricular/confluent WMH | |  |  |  |  |  |
| Solidity^a^ | .29 ± .18 | | .31 ± .20 | .36 ± .20 | 0.03 (-0.17 , 0.23) | -0.01 (-0.15 , 0.13) |
| Convexity | 1.15 ± .18 | | 1.14 ± .18 | 1.17 ± .17 | -0.04 (-0.13 , 0.06) | -0.02 (-0.08 , 0.05) |
| Concavity index | 1.13 ± .13 | | 1.13 ± .24 | 1.08 ± .09 | 0.02 (-0.02 , 0.06) | 0.02 (-0.01 , 0.04) |
| Fractal dimension | 1.68 ± .26 | | 1.67 ± .22 | 1.57 ± .22 | -0.01 (-0.03 , 0.01) | 0.00 (-0.02 , 0.01) |
| Deep WMH | |  |  |  |  |  |
| Eccentricity | .58 ± .15 | | .56 ± .18 | .58 ± .10 | - 1. (-0.05 , 0.09) | -0.02 (-0.08 , 0.05) |
| Fractal dimension | 1.83 ± .20 | | 1.81 ± .35 | 1.88 ± .23 | -0.05 (-0.18 , 0.09) | -0.06 (-0.19 , 0.07) |

Data are represented as means ± SD. Regression analysis were adjusted for age, gender and additionally for natural log transformed WMH volume. Regression beta coefficients are presented with a 95% confidence interval. ^a^Solidity was natural log transformed because of a non-normal distribution.

Supplementary table C: Exploratory post-hoc analyses of the relation between MRI features of SVD (that showed significant between group differences) and frailty components

|  | **WMH volume** | **Concavity index** | **Fractal dimension** |
| --- | --- | --- | --- |
| Weakness | 0.07 (-0.35 , 0.50) | 0.01 (-.02 , 0.05) | 0.19 (-0.35 , 0.72) |
| Weightloss | 0.12 (-0.36 , 0.60) | 0.01 (-0.04 , 0.05) | 0.00 (-0.09 , 0.09) |
| Slowness | 0.65 (0.23 , 1.06)* | 0.04 (-0.01 , 0.08) | 0.12 (0.04 , 0.21)* |
| Mobility | 0.31 (-0.07 , 0.69) | 0.02 (-0.02 , 0.06) | 0.04 (-0.03 , 0.12) |
| Exhaustion | 0.41 (0.00 , 0.82)** | 0.03 (-0.01 , 0.07) | 0.06 (-0.02 , 0.14) |

Exploratory post-hoc analyses of the relation between MRI features of SVD (that showed significant between group differences: total WMH volume and the concavity index and fractal dimension of confluent and periventricular WMH) and frailty components were performed by linear regression analyses corrected for age and gender. WMH volume was additionally corrected for ICV. Data are presented as Beta’s with 95% confidence intervals. WMH volumes were natural log transformed before performing regression analyses. *p =0.003. ** p=0.048


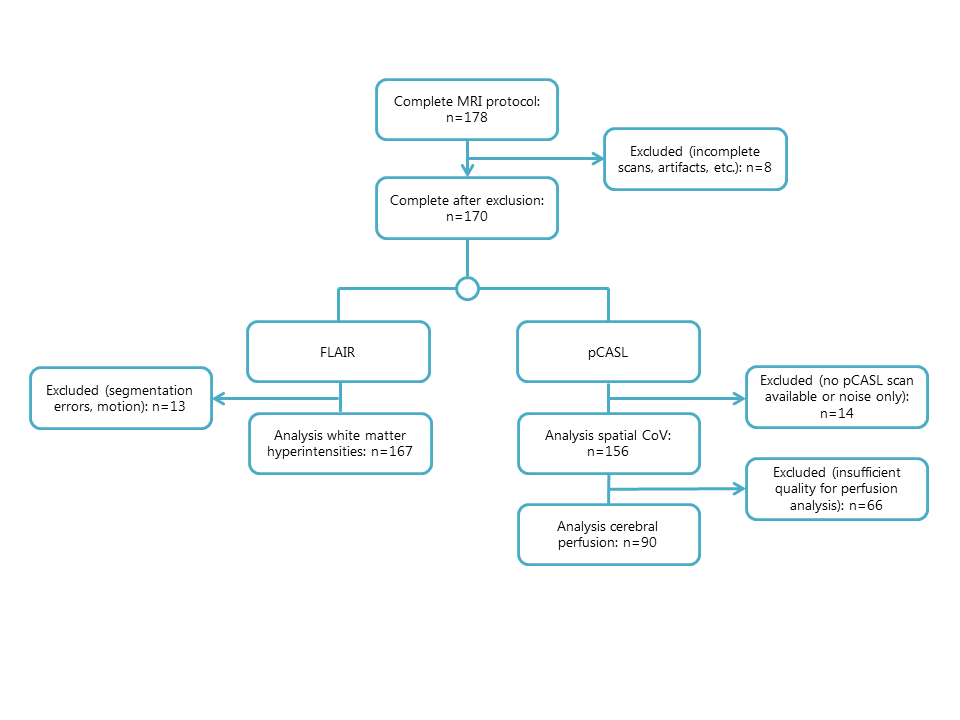


Supplementary figure 1: flowchart of the inclusion of participants
